# Ultrafast Third-Order Nonlinear Optical Response Excited by fs Laser Pulses at 1550 nm in GaN Crystals

**DOI:** 10.3390/ma14123194

**Published:** 2021-06-10

**Authors:** Adrian Petris, Petronela Gheorghe, Tudor Braniste, Ion Tiginyanu

**Affiliations:** 1 National Institute for Laser, Plasma and Radiation Physics, 409 Atomistilor Street, 077125 Magurele, Romania; 2 National Center for Materials Study and Testing, Technical University of Moldova, Stefan cel Mare av. 168, 2004 Chisinau, Moldova; tudor.braniste@cnstm.utm.md (T.B.); ion.tighineanu@cnstm.utm.md (I.T.); 3 Academy of Sciences of Moldova, Stefan cel Mare av. 1, 2001 Chisinau, Moldova

**Keywords:** gallium nitride crystal, third-order nonlinear susceptibility, third-harmonic generation, femtosecond laser pulses, ultrafast nonlinear optical response

## Abstract

The ultrafast third-order optical nonlinearity of c-plane GaN crystal, excited by ultrashort (fs) high-repetition-rate laser pulses at 1550 nm, wavelength important for optical communications, is investigated for the first time by optical third-harmonic generation in non-phase-matching conditions. As the thermo-optic effect that can arise in the sample by cumulative thermal effects induced by high-repetition-rate laser pulses cannot be responsible for the third-harmonic generation, the ultrafast nonlinear optical effect of solely electronic origin is the only one involved in this process. The third-order nonlinear optical susceptibility of GaN crystal responsible for the third-harmonic generation process, an important indicative parameter for the potential use of this material in ultrafast photonic functionalities, is determined.

## 1. Introduction

Gallium nitride (GaN) is a III-V semiconductor with properties that make it an excellent material for high-power, high-voltage, high-frequency electronics [1,2,3]. It is also an important material for electro-optic and integrated-photonic devices [4]. Thus, its wide direct bandgap (*E*_g_ = 3.4 eV) plays a key role in light emitting diodes, laser diodes, and detectors in the blue spectral range of the optical domain [5,6,7]. GaN possesses a large transparency, covering the visible and the near and mid infrared spectral domains [8]. It is chemically stable, has a high optical damage threshold, a weak material dispersion, and a low thermo-optic coefficient [9]. Its wide bandgap makes it also very promising for applications at the telecommunication wavelength of 1550 nm, for which both the two- and the three-photon absorption cannot take place. Electromagnetic interference shielding in an ultra-broad range of frequencies, distributed Bragg reflectors and UV-light driven fluorescent microengines are among the emergent applications of this compound when engineered in three-dimensional nanoarchitectures [10,11,12,13,14,15].

Up until now, the number of studies related to the investigation of the nonlinear optical properties of GaN has still been reduced. Some studies refer to the second harmonic generation in several GaN structures [16,17,18,19]. Other studies refer to the investigation of the third-order nonlinear optical response and to the measurement of the third-order nonlinear optical parameters by Z-scan and four-wave-mixing (FWM) in GaN [20,21,22,23,24,25,26,27,28,29,30]. For visible incident wavelengths, especially when high intensity laser beams are involved, these methods of nonlinear investigation may cause the inclusion of significant two-photon absorption, photo-generated free carriers, and thermo-optic effect contributions in the values of the third-order nonlinear parameters, in addition to the ultrafast electronic one [31]. Only very few studies of the third-order optical nonlinearity of GaN at the telecommunication wavelength of 1550 nm, by four-wave mixing (FWM) with ns pulses [23], and by Z-scan using ultrashort (femtosecond) laser pulses [25] were reported.

To the best of our knowledge, the ultrafast third-order nonlinear response in GaN, excited by femtosecond (fs) laser pulses, has not been measured directly by third-harmonic generation (THG), in particular in the telecommunication spectral range. The THG method allows the direct measurement of the fastest third-order nonlinear optical response of solely electronic origin [31,32,33,34]. At the wavelength of λ = 1550 nm the photon energy is *E*_ph_ = 0.8 eV, which is more than four times lower than the GaN bandgap (*E*_ph_ < *E*_g_/4). Thus, the optical nonlinearity excited in bulk GaN crystal by single-photon absorption at this wavelength is a non-resonant one. Moreover, neither the absorption of two photons nor that of three photons can excite resonant transitions in GaN. Consequently, the third-harmonic generation excited in GaN by fs laser pulses at this wavelength is extremely fast (response time < 10^−15^ s [33]).

In this paper, the ultrafast third-order optical nonlinearity of c-plane GaN crystal, excited by ultrashort (fs) high-repetition-rate laser pulses at 1550 nm wavelength, is investigated for the first time by optical THG in non-phase-matching conditions and the corresponding third-order nonlinear optical susceptibility is determined. The very low average power of the third harmonic beam generated in GaN (~ pW) is measured by image processing, following a method recently introduced by us for the use of a common camera as an ultrasensitive power-meter [35,36].

## 2. Direct Extraction of the Third-Order Nonlinear Optical Susceptibility from the Third-Harmonic Generation

The third-harmonic generation is a nonlinear (NL) optical process in which a third-order NL optical polarization *P^(3)^* is induced in a NL optical material by a fundamental harmonic (FH) laser beam with the frequency *ω*_FH_ *=* *ω*, incident on it [32]:

(1) $$P^{\left(3\right)}=\left(1/4\right)\varepsilon_{0}\chi^{\left(3\right)}E_{\omega }^{3}$$

where *ε*_0_ is the dielectric permittivity of vacuum, *χ*^(3)^ is the third-order NL optical susceptibility of the NL material, associated with the THG process, and *E_ω_* is the electric field of FH beam.

**Figure 1:**
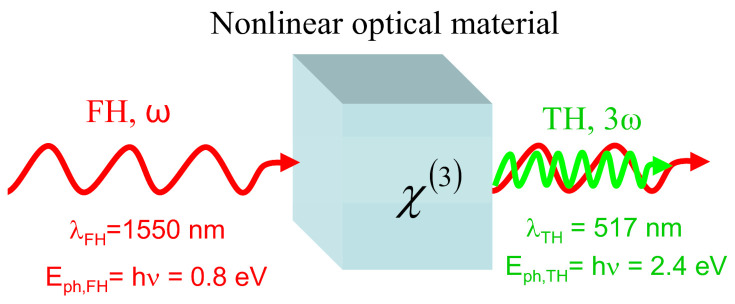
A graphical illustration of the THG process.

This polarization is the source for converting a part of the incident FH beam in a new beam, the TH one, at a three times higher frequency, *ω*_TH_ *=* 3*ω* (or, alternatively at a wavelength λ_TH_ = λ_FH_/3). In contrast to the second-order NL optical process involved in the second harmonic generation, the NL process of THG takes place for any symmetry of the NL optical material and its characteristic NL optical parameter is the third-order NL optical susceptibility, *χ*^(3)^. The THG process is illustrated in Figure 1.

The intensity of the TH beam, was analytically obtained by solving the set of coupled wave equations for the FH and TH waves, considering plane waves, a negligible absorption at both FH and TH wavelengths and a very low conversion efficiency of the FH in TH (the undepleted pump approximation) [32]:

(2) $$I_{3\omega }\left(L\right)=\frac{{\left(3\omega \right)}^{2}{\left|\chi^{\left(3\right)}\right|}^{2}}{16\varepsilon_{0}^{2}c^{4}n_{\omega }^{3}n_{3\omega }}I_{\omega }^{3}\left(0\right)L^{2}\cdot \frac{{\left[\sin\left(\Delta k\cdot L/2\right)\right]}^{2}}{{\left[\Delta k\cdot L/2\right]}^{2}}$$

where *I_3__ω_*(*L*) is the intensity of the TH beam generated inside the NL material, at the exit face of the sample, *I_ω_*(0) is the intensity of the FH beam, at the entrance face of the sample, 3*ω* is the optical frequency of the TH wave, *n_ω_* and *n_3__ω_* are the refractive indices of the NL optical material at the FH and TH frequencies, respectively, *L* is the thickness of the sample, *c* is the speed of light in vacuum, and Δ*k* is the phase mismatch, which is defined as [32]:

(3) $$\Delta k=3k_{\omega }-k_{3\omega }=6\pi (n_{\omega }-n_{3\omega })/\lambda_{\omega }$$

In Equation (3), $k_{\omega }=2\pi \cdot n_{\omega }/\lambda_{\omega }$ and $k_{3\omega }=2\pi \cdot n_{3\omega }/\lambda_{3\omega }$ are the wave numbers of the FH and of the TH, respectively, *λ_ω_* and *λ*_3_*_ω_* being the wavelengths of the respective waves in vacuum.

As revealed by Equation (2), the intensity of the TH beam, *I_3__ω_*(*z*), is proportional to the third power of the FH beam intensity, *I_ω_*(0), and to the square of the *z* coordinate along the propagation path through the NL medium. When the phase mismatch Δ*k* = 0 (phase matching), the square of the sinc function (sinc(x) = sin(x)/x) from Equation (2) equals 1, sinc^2^(Δ*k*⋅z/2) = 1, and the TH beam intensity is maximum. For Δ*k* ≠ 0, the TH beam intensity oscillates along the propagation path as a consequence of the sinc^2^(Δ*k*⋅z/2) function.

The coherence length *L_C_* = *π*/Δ*k* represents the thickness of the NL material for which the argument of the square sinc function from Equation (2) is equal to π/2. When the thickness of the NL sample exceeds *L*_C_, the intensity of the TH decreases significantly, vanishes at 2*L*_C_, and then oscillates as the thickness *L* increases.

The FH wave has been considered as a plane wave in Equation (2). This means a perfectly collimated FH beam. As the intensity of the TH beam is proportional to the third power of FH beam intensity, in order to increase the intensity of the FH beam inside the sample, this beam (considered Gaussian) is usually focused in the NL material with a lens and the plane wave approximation is rigorously fulfilled only in the focal plane of the lens. In this case, in order to ensure the validity of the plane wave approximation, it is important to have a value of the confocal parameter, *b*, much larger than the sample thickness or at least comparable to it.

**Figure 2:**
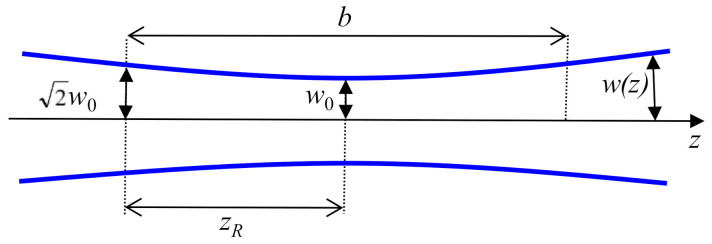
A schematic of the Gaussian beam propagation near the focus of a focusing lens.

The confocal parameter *b* is twice of the Rayleigh length *z_R_* [37]:

(4) $$Z_{R}=\frac{\pi \omega_{0}{}^{2}}{\lambda }$$

in which *w*_0_ is the waist of the focused beam and *λ* is the wavelength of the beam inside the material (the vacuum wavelength divided by the refractive index *n* of the material). The waist *w*_0_ is directly related to the corresponding full width at half-maximum (FWHM) of the beam, $\omega_{0}=FWHM/\sqrt{2\ln2}$ [37]. The Rayleigh length represents the distance between the focal plane of the focusing lens and the plane in which, in propagation, the radius of the beam becomes $\sqrt{2}$ times larger than the waist *w*_0_ of the focused beam (Figure 2).

The third-order NL optical susceptibility, *χ*^(3)^, can be directly computed from the Equation (2), as:

(5) $$\chi^{\left(3\right)}=\sqrt{\left[I_{3\omega }\left(L\right)/I_{\omega }^{3}\left(0\right)\right]/\left[\frac{{\left(3\omega \right)}^{2}}{16\varepsilon_{0}^{2}c^{4}n_{\omega }^{3}n_{3\omega }}\cdot L^{2}\cdot \frac{{\left[\sin\left(\Delta k\cdot L/2\right)\right]}^{2}}{{\left[\Delta k\cdot L/2\right]}^{2}}\right]}$$

by performing a THG experiment in which the intensities of the incident FH beam and of the generated TH beam are measured and the thickness of the investigated NL material and its refractive indices at the two involved wavelengths are known.

## 3. Experimental Details of the Third-Harmonic Generation in GaN Crystal

### 3.1. Experimental Setup

**Figure 3:**
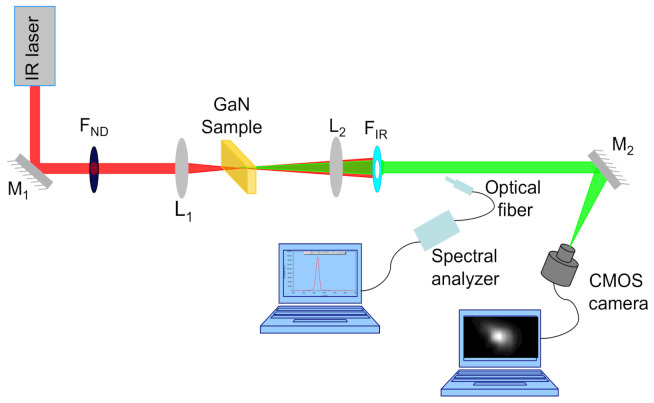
Schematic of the experimental setup used for THG in GaN crystal.

The experimental setup used for THG experiments in GaN crystal is schematically shown in Figure 3.

The optical THG experiments were performed using an Er-doped fiber laser (FemtoFiber Scientific FFS, TOPTICA Photonics AG, Munich, Germany), which generates ultrashort pulses with pulse duration *τ* = 120 fs at the wavelength *λ*_FH_ = 1550 nm, with a repetition rate *f*_rep_ = 76 MHz and the maximum average power *P*_FH,av,max_ = 228.3 mW as the source of the FH beam.

The GaN sample is placed between the lenses L_1_ (5 cm focal length) and L_2_ (3.5 cm focal length) mounted on micrometric translation stages for the fine tuning of their positions relative to the sample. The lens L_1_ focuses the FH beam down to a spot of 26 μm (FWHM) on the sample placed in its focal plane.

The very low average power (~pW) of the TH beam generated in non-phase-matching conditions was measured using a common CMOS camera (Thorlabs, DCC1545M), without objective, as a power-meter, following a calibration method introduced by us and briefly described below [35]. The photosensitive array of the camera used by us (6656 μm * 5325 μm) consists of 1280 px * 1024 px, with the pixel size of 5.2 μm. With the lens L_2_, which collects the entire TH beam, the spot size of TH beam generated inside the sample is adjusted relative to the size of the camera photosensitive array, ensuring high grey levels on the camera pixels illuminated by TH beam, yet avoiding their saturation.

**Figure 4:**
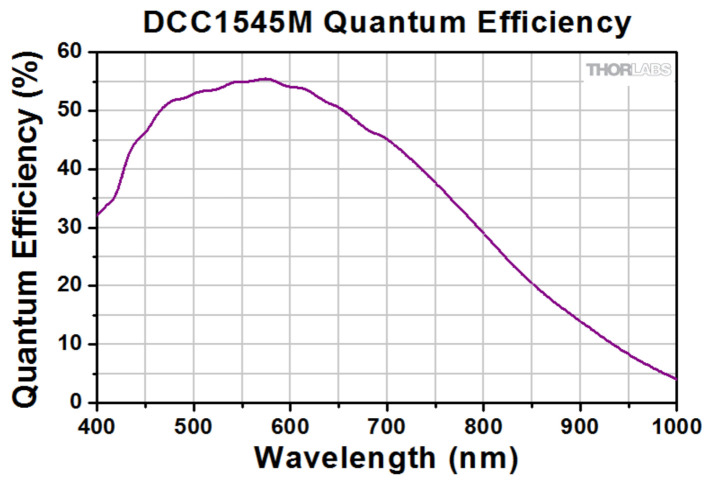
Quantum efficiency of the DCC1545M CMOS camera.

The intensity of the FH beam incident on the sample is adjusted by changing only its power with neutral filters (F_ND_) calibrated at the FH wavelength. The FH beam, which is exiting from the sample, is cut off with two IR blocking filters (F_IR_) with known transmission at the wavelength of the TH. An additional filtering of IR radiation is provided by the camera itself, as it is possible to see in the Figure 4, in which the quantum efficiency of the photosensitive array of this camera is shown, available from https://www.thorlabs.com (accessed on 14 May 2020).

The average powers, *P*_FH,av_, of the incident FH beam have been measured with an optical power-meter (Coherent, FIELDMAXII-TOP with OP-2 IR sensor), before the focusing lens L_1_.

The CMOS camera is connected to a computer that acquires the images of the TH beam spot, generated at different incident intensities of the FH beam. The average power of the TH beam is computed by processing these images, following a method recently introduced by us for the use of a common camera as an ultrasensitive power-meter [35].

The wavelength of the TH beam is measured with a fiber optic spectrometer (Ocean Optics model HR4000CG-UV-NIR) connected to the computer.

### 3.2. Measurement of Very Low Optical Powers Using a Camera as Power-Meter

The method used for the measurement of very low optical powers using a common camera as a very sensitive power-meter is described in detail elsewhere [35]. Here, we will mention briefly the principle and several advantages of this method.

The measurement of the optical power with a camera is based on the fact that each pixel of the camera’s photosensitive array can be considered as a power-meter of micrometric size that generates an electric signal proportional to the incident optical signal in the linear operation regime. By cumulating the response of all pixels and using a calibration procedure developed by us, the incident optical power can be measured down to extremely weak (fW) light levels, as was demonstrated in [35].

When using the camera, it is possible to see the light distribution inside the spot of the measured light beam, its evolution during the experiment, and it is easier to make more obvious the connection of the measured power change with an eventual spatial light distribution variation. Different ranges of incident optical powers can be measured by changing the exposure time of the camera. When the incident optical power is decreased, the size of the beam spot on the camera’s chip can be decreased with the lens L_2_, maintaining the linear operation regime with no saturated pixels. By collecting the signal from a software generated window that contains the pixels illuminated by the measured beam, a large signal-to-noise ratio can be maintained even at very low incident powers. Related to this aspect, at a commercial power-meter the electrical signal is generated by the entire sensor area, no matter how small the size of the measured spot is.

This method also provides an additional advantage in the measurement of very low powers of the TH beam generated in non-phase-matching conditions. Thus, only the signal from the pixels that are inside the software generated window, which surrounds the image of the small size TH beam spot on the photosensitive array, is collected. Therefore, the influence of residual IR light of the FH beam, which, eventually, is not completely filtered by the IR blocking filters and by the camera itself (as previously mentioned) is highly reduced being imaged by the lens L_2_ in another plane than the TH beam spot is.

### 3.3. The Investigated Sample

The investigated sample is a commercial free-standing c-plane uniaxial HVPE grown n-GaN crystal (LUMILOG Saint-Gobain) with a Wurtzite-like crystal structure and with the thickness *L* = 400 μm along the *c*-axis. The crystal is of (0001)-orientation with virgin Ga-face and polished N-face. The density of threading dislocations is in the range of (1–2) × 10^7^ cm^−2^. Previous experiments revealed fine spatial modulation of the electrical conductivity in this crystal attributed to instability in the growth direction [38,39].

**Figure 5:**
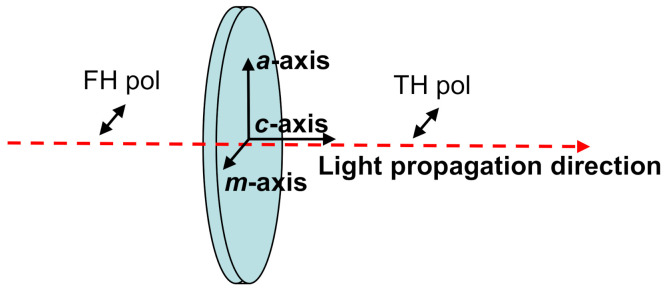
Schematic representation of crystallographic *a*, *c*, and *m* axes relative to the investigated GaN sample plane. The light propagation direction and its polarization are also shown.

The directions of the crystallographic *c*, *a, m* axes relative to the sample plane are shown in Figure 5. The GaN sample was placed in the experimental setup oriented as it is shown in the Figure 5, with the light propagation direction along the *c*-axis and orthogonal to the *m* and *a* axes. The polarization of both IR FH and green TH beams is horizontal, orthogonal to the *c* and *a* axes, and parallel to the *m*-axis (Figure 5). In this experimental configuration, the *n*_o_ refractive index of the GaN sample (positive uniaxial crystal, *n*_e_ > *n*_o_) is accessed by both FH and TH light beams. At normal incidence of the FH beam, with this particular geometry of the GaN sample, the *n*_e_ refractive index of GaN cannot be accessed, independently on the rotation around the *c*-axis of the sample or, which is equivalent, of the FH beam polarization.

The values of the refractive indices (*n_ω_* and *n_3__ω_*) of the NL material at the FH and TH frequencies, respectively, were calculated using the Sellmeier equation [40] for the ordinary refractive index, *n_o_*, given by the Equation (6):

(6) $$n_{0}^{2}\left(\lambda \right)=5.346+\frac{0.1377\;\mu m^{2}}{\lambda^{2}-0.1573^{2}\;\mu m^{2}}+\frac{0.01492\;\mu m^{2}}{\lambda^{2}-0.3524^{2}\;\mu m^{2}}$$

where *λ* is the wavelength (in μm). The ordinary refractive indices of the GaN crystal, at the FH and TH wavelengths, determined from Equation (6), are: *n*_ω_ = 2.32604 at *λ*_ω_ = 1550 nm and *n*_3__ω_ = 2.45316 at *λ*_3__ω_ = 517 nm. Using these values of the refractive indices, we determined the value of the phase mismatch and the value of the coherence length, *L*_C_, for the investigated sample: $\left|\Delta k\right|$ ≅ 1.5458 × 10^4^ cm^−1^, *L_C_* ≅ 2 μm. The Rayleigh length, given by Equation (4), for our investigated sample is *z_R_* ≅ 980 μm. The corresponding confocal parameter is *b* ≅ 1960 μm, which is more than four times higher than the sample thickness. So, we can assume a satisfactory validity of the plane wave approximation along the propagation path of the FH beam through the sample. The assumption of negligible absorption in GaN, at both FH and TH wavelengths, is well fulfilled [25,41,42] as well as the assumption of the undepleted pump approximation due to the very low conversion efficiency (<10^−8^ in our experiment) of the FH in TH (no phase-matching), for which the intensity of the TH beam (Equation (2)) was analytically obtained.

## 4. Results and Discussion

**Figure 6:**
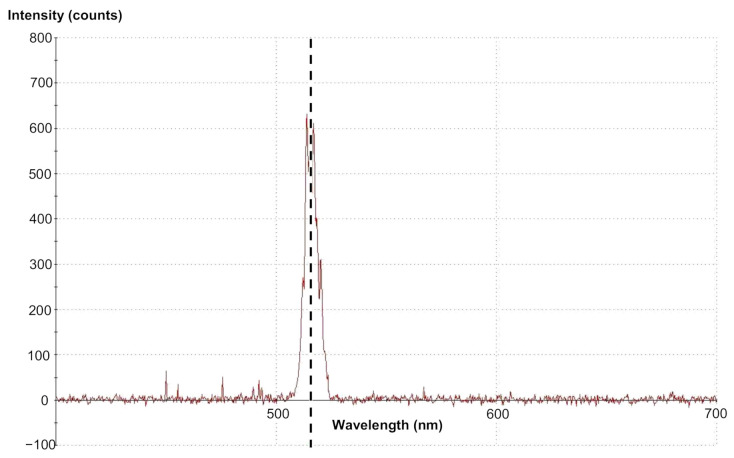
The spectrum of the TH wave generated in GaN crystal.

The spectrum of the TH beam generated in GaN sample is shown in Figure 6. The black dashed line shown in this figure is at the wavelength *λ*_TH_ = 517 nm.

We experimentally determined the average power of the FH beam (~mW) by direct measurement with a power-meter, and of the TH beam (~pW), by processing the images of the TH beam spot acquired with the CMOS camera specially calibrated to measure very low optical powers [35]. The analytical expression for the conversion from the average power *P_av_* of a train of ultrashort laser pulses with Gaussian transversal spatial profile and sech^2^ temporal profile to the peak intensity *I_peak_*, based on [37], is:

(7) $$I_{peak}=2\cdot P_{peak}/(\pi w_{0}^{2})=2\cdot \left(0.88\cdot \frac{P_{\;av}}{\tau \cdot f_{rep}}\right)/(\pi w_{0}^{2})$$

where *w*_0_ is the waist of the laser beam, *τ* is the laser pulse duration, and *f_rep_* is the repetition rate of laser pulses. The peak intensities of the TH beam for several peak intensities of the FH beam have been calculated from the corresponding TH average powers, in order to determine the dependence *I_3__ω_*(*L)* = *f* (*I_ω_*(0)), where *I_3__ω_*(*L)* ≡ *I*_TH,peak_(*L*) and *I_ω_*(0) ≡ *I*_FH,peak_(0). From the fitting of the experimental dependence *I_3__ω_*(*L)* = *f* (*I_ω_*(0)) with a third grade polynomial, *I_3__ω_* = *C*_THG_ · *I_ω_*^3^, we determined the coefficient *C*_THG_ *= I_3__ω_/I_ω_*^3^, then from the Equation (5) we calculated the third-order NL optical susceptibility corresponding to the THG process, *χ*^(3)^, in the c-plane GaN crystal. It characterizes the ultrafast nonlinear response of solely electronic origin in this material excited with ultra-short laser pulses at the wavelength of 1550 nm.

The maximum value of the average power of the FH beam, measured with the optical power-meter, was *P*_FH,av,max_(0) = 228.3 mW. The corresponding *I*_FH,peak_(0) of the FH laser pulses, considered with a Gaussian transversal spatial profile and sech^2^ temporal profile is *I*_FH,peak,max_(0) = 2.29 GW/cm^2^. The range of the incident FH peak intensities, considered in our experiments, is *I*_FH,peak_(0) = (0.99 ÷ 2.29) GW/cm^2^.

The average powers of the TH beam, *P*_TH,av_(*L*) have been measured with the calibrated CMOS camera used as a very sensitive power-meter [35]. Each of these powers has been determined in two ways: by considering the entire photosensitive array of the camera used (1280 px * 1024 px = 1,310,720 pixels) as well as considering a software generated window of 200 px * 200 px (40,000 pixels), surrounding the TH beam spot, which represents only 3% of the surface of the photosensitive array of the camera. When measuring very low optical powers by the method described above, the use of a software generated window that surrounds the measured beam spot is preferable in terms of accuracy, as it is discussed in detail in [35]. The necessary corrections for Fresnel reflections of the FH beam on lens L_1_ and of the TH beam on lens L_2_ have been made. The reflection losses of the FH beam at the air–GaN interface (15.9%) and of the TH beam at the GaN–air interface (17.7%) have been taken into account. The attenuation of TH beam by the two IR blocking filters, F_IR_, has been also taken into account.

**Figure 7:**
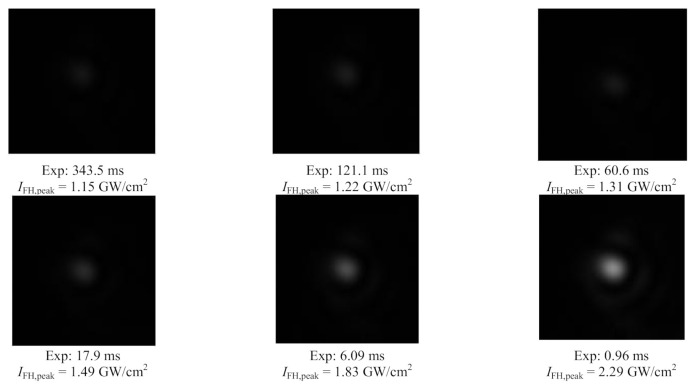
Images of the TH beam spot generated in GaN crystal, framed in a 200 px ∗ 200 px window at several peak intensities of the incident FH. The exposure times corresponding to the recordings are mentioned.

In Figure 7 we show one set of images of the TH beam spot generated in the GaN crystal, considering the software generated window of 200 px × 200 px, from which the corresponding average powers of TH are determined for the different considered incident intensities of FH beam. The image of TH beam spot on the camera, corresponding to the lowest considered intensity *I*_FH,peak_(0) = 0.99 GW/cm^2^ of the FH beam, is not shown in the Figure 7, as it is difficult to be seen by the naked eye due to the very low grey levels of its pixels.

From the processing of the recorded images, one set considering the entire photosensitive array of the camera (1280 px * 1024 px) and another set considering the software generated window of 200 px * 200 px, the peak intensities of the generated TH beam were determined and the corresponding experimental dependencies of the TH intensity on the FH intensity, *I*_TH,peak_(*L*) = *f* (*I*_FH,peak_(0)), were obtained.

**Figure 8:**
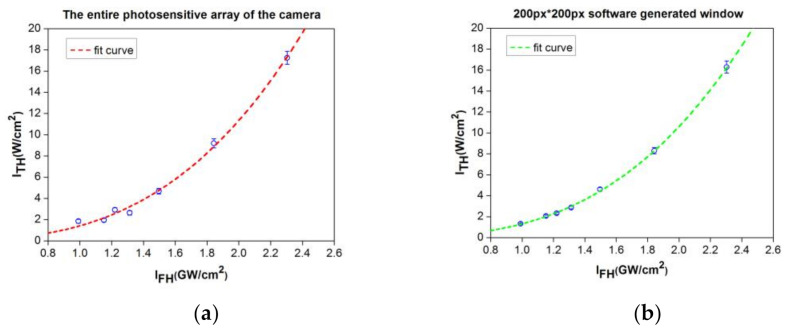
The experimental curves *I*_TH,peak_(*L*) = *f* (*I*_FH,peak_(0)), considering the entire photosensitive array of the camera (**a**), and a 200 px * 200 px software generated window surrounding the measured beam spot (**b**), respectively.

By comparing the experimental points and their distribution relative to the fit with a third grade polynomial, shown in the Figure 8a,b, it is clear, as it was expected, that the results are better when considering the software window than when considering the entire area of the camera sensor, with a lower spread of the experimental points against the fit curve.

The third-order NL optical susceptibility corresponding to the third-harmonic generation process in the investigated c-plane GaN crystal with the FH beam polarization parallel to the *m*-axis, determined from the experimental data, is *χ*^(3)^ = (1.30 ± 0.15) × 10^−20^ m^2^/V^2^, which is obtained when the software generated window is taken into account. To the best of our knowledge, this is the first direct measurement, by THG, of the ultrafast optical nonlinearity of solely electronic origin in c-plane GaN crystal.

The magnitude of the *χ*^(3)^ obtained by us is in qualitative agreement with several values of the third-order NL susceptibility obtained by other experimental techniques, as Z-scan and wave mixing, at similar wavelengths. Thus, in 2019, in [25] the nonlinear refractive index of a ~10 μm GaN layer grown epitaxially on a sapphire substrate was measured by Z-scan technique performed with 120 fs laser pulses in the wavelength domain (1550 nm ÷ 550 nm), and the value obtained at 1550 nm was *n*_2_ ≈ 90 × 10^−20^ m^2^/W. The corresponding value of the nonlinear optical susceptibility responsible for this intensity dependent refractive nonlinearity is proportional to the nonlinear refractive index *n*_2_ [33] and has the magnitude *χ*^(3)^ ≈ 1.71 × 10^−20^ m^2^/V^2^. In 2018, in [23] the nonlinear refractive index *n*_2_ was measured in a GaN ridge waveguide by FWM with ~30 ns laser pulses at the telecom wavelength of 1550 nm and the estimated value was *n*_2_ ≈ 3.4 × 10^−18^ m^2^/W. The corresponding value of the nonlinear susceptibility is *χ*^(3)^ ≈ 6.45 × 10^−20^ m^2^/V^2^. We have to mention that this comparison of the value of *χ*^(3)^ obtained by us with the values from [23,25] is only a qualitative one, as different NL optical processes are involved in the nonlinear optical response measured in these techniques [23,25,31].

## 5. Conclusions

The fastest third-order optical nonlinearity of solely electronic origin was measured for the first time, to the best of our knowledge, in c-plane GaN crystal by the third-harmonic generation excited by high-repetition-rate ultrashort laser pulses (fs) at the telecommunications wavelength of 1550 nm. The very low average power of the third harmonic beam generated in GaN (~pW) in non-phase-matching conditions was measured by image processing, following a method recently introduced by us for the use of a common camera as an ultrasensitive power-meter. The third-order nonlinear optical susceptibility, *χ*^(3)^, responsible for the nonlinear optical process of frequency conversion was directly determined in c-plane GaN crystal from the third-harmonic generation experimental results. Its value, *χ*^(3)^ = (1.30 ± 0.15) × 10^−20^ m^2^/V^2^, is in qualitative agreement with several values of the third-order nonlinear optical susceptibility obtained at similar wavelengths by other experimental techniques (Z-scan, four-wave mixing), in which different nonlinear optical processes of the third-order are involved. The obtained results are important for the potential use of GaN in ultrafast photonic functionalities.

## Data Availability

Data underlying the results presented in this paper are not publicly available at this time but may be obtained from the authors upon reasonable request.
